# Using functional Magnetic Resonance Imaging to differentiate between healthy aging subjects, Mild Cognitive Impairment, and Alzheimer’s patients

**Published:** 2010

**Authors:** Mohammad Ali Oghabian, Seyed Amir Hossein Batouli, Maryam Norouzian, Maryam Ziaei, Hajir Sikaroodi

**Affiliations:** a Medical Physics and Biomedical Engineering Department, Tehran University of Medical Sciences, Tehran, Iran; b Radiation Medicine Engineering Department, Shahid Beheshti University, Tehran, Iran; c Neurologist, Memory and Behavioral Neurology Department, Tehran University of Medical Sciences, Tehran, Iran; d Psychology Department, Shahid Beheshti University, Tehran, Iran; e Neurologist, Department of Neuro-Vascular Diseases, Shariati Hospital, Tehran, Iran

**Keywords:** FMRI, Default Mode Network, Alzheimer’s, Mild Cognitive Impairment, Resting-State

## Abstract

**BACKGROUND:**

Alzheimer’s disease is the most common form of dementia which is still difficult to be differentiated from other types of brain disorders. Moreover, Mild Cognitive Impairment refers to the presence of cognitive impairments that is not severe enough to meet the criteria of Alzheimer’s, and its diagnosis in early stages is so critical. There is currently no distinct method available for diagnosing Alzheimer’s or Mild Cognitive Impairment, and their diagnosis needs a combination of different methods and assessments.

**METHODS:**

The aim of this study was to evaluate the effectiveness of functional Magnetic Resonance Imaging (fMRI) in differentiating between Alzheimer’s, Mild Cognitive Impairment (MCI) and healthy aging. To prove fMRI’s ability, resting-state brain activation patterns between these three groups of subjects were compared using Independent Component Analysis (ICA) algorithm. Forty age- and sex-matched subjects, 15 elderly, 11 MCI and 14 Alzheimer’s subjects were examined.

**RESULTS:**

The results showed that during a certain resting-state session, healthy aging brain benefits from larger area and greater intensity of activation (compared with MCI and Alzheimer’s group) in Posterior Cingulate Cortex (PCC) region of the brain, as part of Default Mode Network.

**CONCLUSIONS:**

This difference in activation pattern can be used as a diagnostic criterion in using fMRI for differentiating between Alzheimer’s Disease (AD), MCI and healthy aging.

Alzheimer’s disease (AD) is a progressive brain disorder which in severe stages causes thinking and memory abilities to become seriously damaged.^1^ Alzheimer’s accounts for 60% to 80% of dementia cases, and that’s why it is the most common form of dementia. The problems with memory, thinking and behavior may become severe enough to affect work, daily activities and social life.^2^ Alzheimer’s progresses in time and will finally cause death. It is reported that about 26.6 million people all around the world were afflicted by Alzheimer’s in 2006 and this number may quadruple by 2050.^3^,^4^ Although there is no definite cure or proven treatment available to slow down Alzheimer’s progression, there are a few number of medications and treatments currently available that may help improve the mental functions of Alzheimer’s patients.^5^ If these medications and treatments are given in the early stages of the disease, they may be highly effective in enabling people to carry out their daily activities, consequently living independently for a longer period of time.^5^ To obtain this goal, scientists are trying to find practical methods for early diagnosis of AD. Nowadays, there is no definite method for diagnosing Alzheimer’s, so a variety of assessments and laboratory measurements are being used by doctors to diagnose Alzheimer’s. Advanced medical imaging methods such as Computed Tomography (CT), Magnetic Resonance Imaging (MRI), Single Photon Emission Computed Tomography (SPECT) or Positron Emission Tomography (PET) can be used to help experts in the process of diagnosing Alzheimer’s.^6^,^7^ Moreover, fMRI is a functional medical imaging method, which seems to be able to contribute or improve the process of early diagnosis of Alzheimer’s disease.

Mild Cognitive Impairment (MCI, also called incipient dementia, or isolated memory impairment) is a diagnosis given to patients who have cognitive impairments, more severe than expected for their educational level and age, and which does not interfere significantly with their daily life and activities.^8^ In fact, MCI is a transition stage between the cognitive impairments of healthy aging subjects and the more serious problems caused by Alzheimer’s disease.^9^ This disorder affects many areas of thought and action, such as language, attention, reasoning, judgment, reading and writing; although Mild Cognitive Impairment in the most common form causes memory problems.^9^ The prevalence of mild cognitive impairment increases with age. The prevalence is 10% in those aged 70-79 years and 25% in those aged 80-89 years.^10^ Subjects with MCI are of major clinical importance because they have an increased risk of developing Alzheimer-type dementia.^11^ It is of major importance to identify subjects with MCI who might become demented, in order to give them a prognosis and allow starting treatment in an earlier phase than is possible now. Diagnosing MCI today needs a combination of several clinical methods, such as survey in patient’s clinical history, neurological exam and at least a brief neuropsychological evaluation. Its diagnosis might also need further testing, including blood-work and brain imaging.^12^ FMRI is one of those brain imaging methods, which is going to be evaluated here.

Functional Magnetic Resonance Imaging is a type of MRI scan which measures brain’s haemodynamic response related to a neural activity. This technique uses BOLD (Blood Oxygen Level Dependant) effect in order to show the pattern and intensity of activation in human’s brain. It has some advantages including low invasiveness, lack of radiation exposure, and relatively wide availability.^13^ FMRI data acquisition and analysis can be done either using model-based or non modelbased methods.^14^ In the model-based method, an external stimulus is presented to the subject and brain’s haemodynamic response will be aligned to it. In the non model-based method, no external stimulation exists and therefore brain’s activation in this form is not in accordance with any predefined stimulation pattern. This state of the brain is so called the “restingstate”, since the patient is not voluntarily doing any function and all of the observed functions at the moment are caused by internal stimulations. These kinds of stimulations might be the result of passive or routine functions of the brain such as mental functions, controlling the conditions, supervising the environment, registering the feelings and emotions and other forms of undirected thoughts.^15^ The network of these brain functions which are activated when the subject is at resting-state is so called “Default Mode Network”.^16^

The default-mode network (DMN) consists of some specific brain regions, whose activity is important during resting-state and diminishes during cognitively demanding complex tasks.^17^ This network is mostly active when subjects are not concentrating on their external environment.^18^ Consequently, the main characteristic of DMN is its existence during rest and its disappearance or weakening by confronting the subject to the stimuli (deactivation). The default-mode network is mostly observable in Posterior Cingulate Cortex (PCC)^16^ and in Prefrontal areas.^19^ It was shown that the activation pattern in DMN is different in Alzheimer’s patients compared with healthy aging.^15^ This different resting-state activation would be a good biomarker for differentiating Alzheimer’s patients from healthy aging.

The aim of this study was to evaluate the effectiveness of fMRI for differentiating between Alzheimer’s disease, MCI and healthy aging. Although there are some researches currently available about diagnosing AD from healthy aging by using fMRI, they are different with the present one in two aspects. The first is that, to date, no study has considered these three groups of subjects simultaneously for imaging, and that is the concentration of this study. The second point also emphasizes on the resting-state session. The subjects in the present research are assessed under a pure resting-state condition.

To obtain the goal, fMRI activation patterns in resting-state were compared between the three groups of subjects using Independent Component Analysis (ICA) algorithm. The results of this evaluation enable using fMRI for diagnosing Alzheimer’s or even predicting MCI, to start medication for the patients in the earlier stages of the disease.

## Methods

### 

#### Subjects

A total number of 40 subjects, including 15 old subjects (8 males and 7 females, 60-85 years old, mean 70), 14 Alzheimer’s subjects (8 males and 6 females, 70-84 years old, mean 76) and 11 MCI subjects (5 males and 6 females, 61-78 years old, mean 68), were examined in this study. The inclusion criteria required all subjects to be right-handed, non-smokers, and without any special brain disease and sign of hypertension or psychological symptoms such as depression. All subjects were examined clinically by both neurologists and psychologists and the criteria were also checked by interviewing subjects or their relatives. Besides, according to GDS test (Global Deterioration Scale), Alzheimer’s and MCI patients were categorized to 7 categories. The first category shows healthy subjects and the 6^th^ and 7^th^ ones stand for severe Alzheimer’s. Meanwhile, the middle categories show different stages of MCI. According to this clinical test, all Alzheimer’s patients had GDS score of 6 or 7, all MCI patients had GDS of 3 and 4 and all healthy aging subjects had GDS of 1. It’s worth mentioning that among these 40 subjects, 11 imaging data were omitted from final analysis, due to some reasons such as inability of patients to do what was expected during resting-state or due to some technical problems such as high value of distortion in their imaging data. Hence the final number of subjects for analysis reduced to 10 healthy aging, 9 MCI and 10 Alzheimer’s subjects. (Table 1)

**Table 1:** Demographic information about subjects

Subjects	Control	MCI	Alzheimer’s
Number of subjects	15	11	14
Male/Female	8/7	5/6	8/6
Mean age	70 ± 7.8	68 ± 6.5	76 ± 4.6
GDS score	1	3, 4	6, 7
Final number of subjects	10	9	10

#### Experiment

During fMRI, and to create resting-state conditions, the subjects were asked to lie back relaxed in the MR scanner, with their eyes closed and not to think about anything, do any function or move their head or limbs.

#### Imaging

The MRI system was a 1.5-Tesla GE® Signa scanner. A T1-Weighted Spin-Echo sequence was used to acquire high-resolution structural maps of the brain (Axial, TR = 400 ms, TE = 9 ms, Flip Angle = 90°, voxel size = 1×1×6 mm). FMRI data were obtained with the same dimension and orientation of the structural images, employing a Gradient Echo/Echo planar imaging (EPI) protocol (Axial, TE = 60.3 ms, TR = 3125 ms, Flip Angle = 90°, Field of View = 22 cm^2^, Number of Slice = 15, Slice Thickness = 6 mm, Spacing= 0 mm, Bandwidth = 15.62 KHz, voxel size = 4×4×6 mm). Due to the hardware limitations (maximum 32 time-points with 15 brain slices in the MR scanner), the EPI sequence had to be repeated to acquire the reasonable number of image volumes for data analysis. Each EPI sequence lasted for 100 seconds, and by repeating the sequence, 64 functional image volumes (time points) were obtained finally.

#### Functional Data Analysis

The functional analysis was performed using spatial Independent Component Analysis (ICA) algorithm. ICA is a statistical method that can be used to discover hidden factors (sources or features) from a set of measurements or observed data such that the sources are maximally independent and their probability distribution functions are non-Gaussian.

**Figure 1:**
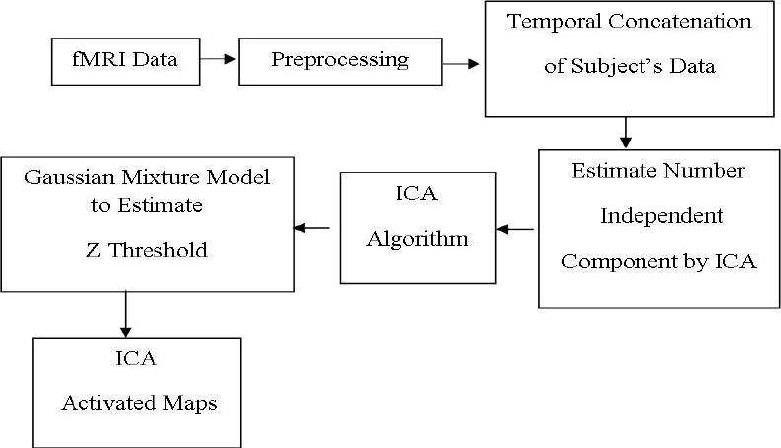
The block diagram of ICA analysis

This algorithm has been used previously in the analysis of resting-state fMRI data^20^,^21^ to extract the brain activated regions’ pattern, since it is a non model-based imaging method. Melodic (Multivariate Exploratory Linear Optimized Decomposition into Independent Components) tool of the FSL (FMRIB Software Library, version 4.1) software was used to analyze the functional imaging data of the three groups of subjects (healthy old, MCI and Alzheimer’s), in the form of Multi-Session temporal concatenation group PICA (Probabilistic Independent Component Analysis) algorithm.^22^ The block diagram of the data analysis is shown in figure 1.

Before data analysis, some preprocessing steps were performed. The preprocessing consisted of seven stages: 1) head motion correction using MCFLIRT (Motion Correction using FMRIB’s Linear Image Registration Tool), 2) slice-timing correction using Fourier-space time-series phase-shifting, 3) mean intensity normalization of the entire 4D dataset by a single multiplicative factor, 4) spatial smoothing using a Gaussian kernel of FWHM of 5 mm, 5) brain extraction to remove non brain tissues using Brain Extraction Tool (BET, Version 1.1), 6) high-pass temporal filtering (Gaussian-weighted least-squares straight line fitting, with sigma = 100 s) and 7) Gaussian low-pass temporal filtering (sigma = 2.8 s).

For estimating the number of independent components, Minimum Description Length algorithm (MDL) was used^23^ which assumes a penalized form of likelihood function and minimizes it to estimate the number of independent sources. All extracted components were transformed to Z-space in order to have a zero mean and unit variance. In the next step, a Gaussian Mixture Model^24^ was used to find a suitable threshold for each independent component. Final ICs were extracted by implementing this threshold on the probability component maps. After functional analysis all the functional images were registered to their anatomical T1 images.

Separate ICA group analysis was used in the form of Multi-Session Temporal Concatenation for the three groups of subjects. Of course, in this from, a standard image from FSL standard library was used to overlay the activation components on it. MIN152_T1_2mm_brain standard image was used for visualization of activation components in group analysis. Nonetheless, all the analysis parameters were the same between the three groups, and hence the comparison of the results is valid.

It’s worth to be mentioned that fMRI data analysis by MELODIC tool reveals several components of brain activation, which may be related to visual cortex, auditory cortex, Default Mode Network, memory, or even motor cortex. However among all output components of brain activation, just the components which show some parts of Default Mode Network were selected. As a matter of fact, in this project, the most important region of interest included in the DMN was Posterior Cingulate Cortex (PCC). Additionally, since all the components are spatially independent from each other, selecting PCC’s component among all is not a difficult task, and just needs some knowledge about brain Neuroanatomy.

## Results

In order to compare statistically the three groups of patients and controls, activation cluster size and activation cluster mean Z value were used.

**Figure 2:**
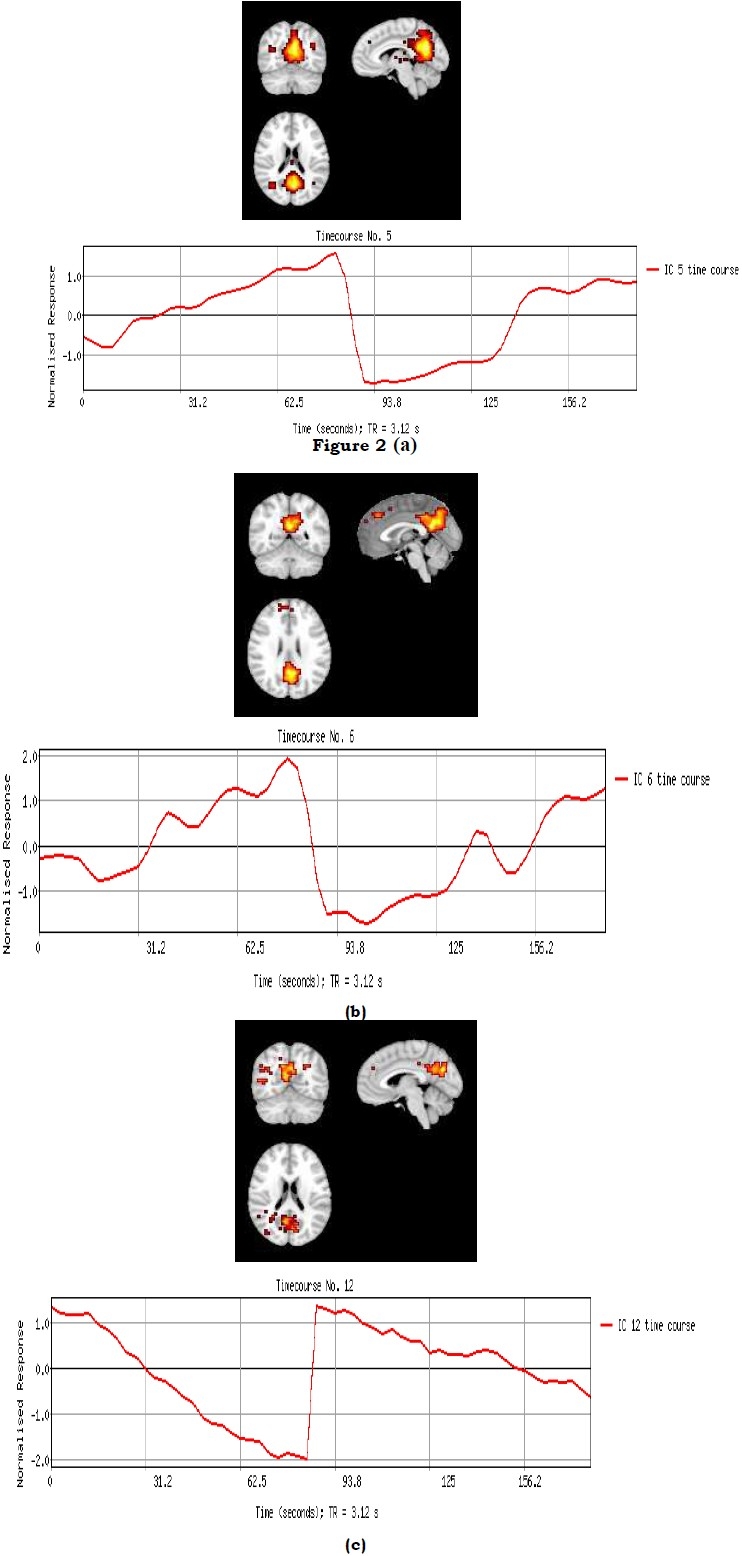
Activation pattern with activation time-course in Posterior Cingulate Cortex during resting-state (a): Healthy Old; (b):MCI; (c):Alzheimer’s

Figure 2 shows the activated region (PCC) in the three groups of Alzheimer’s, MCI and healthy aging, in Axial, Sagittal and Coronal view, plus their activation time-courses.

The statistical analysis results of Posterior Cingulate activation for the three groups are provided in the table 2.

**Table 2:** Statistical analysis results for PCC region in the three groups

Statistical analysis results	Healthy old	MCI	Alzheimer’s
Min intensity	3.375	3.278	3.203
Max intensity	10.427	7.554	6.251
Number of active voxels in group analysis	532 ± 208.45	372 ± 48.4897	235 ± 110.67
Mean Z value of activation cluster	5.606 ± 0.957	4.479 ± 0.2785	4.019 ± 0.768

To have a better comparison between the statistical results of the three groups, table 3 provides p values for the three groups, for both activation cluster size and activation mean Z value.

**Table 3:** Comparison between groups by p value

Comparison by p value	Significance of difference in mean cluster size between groups	Significance of difference in mean Z threshold between groups
Healthy aging and MCI	0.0097	0.0010
Healthy aging and AD	0.0051	0.0023
MCI and AD	0.0168	0.3132

As can be seen in table 2, activation cluster size in healthy aging group is greater than MCI group (532 > 372, p = 0.0097) and than AD group (532 > 235, p = 0.0051), and the differences are significant. It can also be observed that activation cluster size in MCI group is significantly greater than AD’s, (372 > 235, p = 0.0168).

On the other hand, to have a comparison on cluster mean Z value, it is observed that mean Z value in healthy aging is greater than MCI (5.606 > 4.479, p = 0.0010), and Alzheimer’s (5.606 > 4.019, p = 0.0023). Meanwhile, although mean activation intensity in MCI is greater than AD, their difference is not significant (4.479 > 4.019, p = 0.3132).

## Discussion

In this work, a brain activation network called “Default Mode Network” was studied, in order to evaluate the efficiency of fMRI for differentiating between healthy aging, MCI and Alzheimer’s subjects. It was showed that the activation cluster size and intensity in PCC region of Alzheimer’s group is smaller in comparison to MCI and is smaller in MCI compared with healthy aging subjects. This was found to be 532 > 372 > 235 for cluster size and 5.606 > 4.479 > 4.019 for activation intensity, all with p value < 0.05, except for 4.479 > 4.019.

Several studies has been done up to now about using fMRI for diagnosing Alzheimer’s disease. In a study, scientists have used fMRI to diagnose any impairment in visual cortex of Alzheimer’s patients, compared with healthy aging.^25^ In another study, fMRI has been used to diagnose impairments in learning ability of Alzheimer’s patients.^26^ Another research has been done on using fMRI to diagnose Alzheimer’s patients’ problems in decision making tasks.^27^ In more recent studies, it has been revealed that some specific brain regions are active during resting-state constituting the Default Mode Network.^16^ Although several studies have been done using fMRI to diagnose Alzheimer’s from healthy aging, none included MCI and AD subjects simultaneously, and few of them were performed during pure resting-state as it was done in the present study. Although in a model-based fMRI study,^15^ a simple sensory-motor processing task was used to diagnose AD patients from healthy aging subjects during resting-state brain activity, but the subjects were not actually at rest during imaging and they performed a simple sensory-motor task.

In the current research, initial goal was to find the Default Mode Network active in brain during resting-state, in all the three groups of subjects. It was shown that all the three groups showed some kinds of activation in the Posterior Cingulate region of the brain, as the most important region included in DMN. This region of the brain (PCC) has been shown active during resting-state for healthy aging and Alzheimer’s subjects in previous studies as well,^20^,^25^,^28^ and so the present results, in this regard were in accordance with them.

The second part of this research was to find impairments in resting-state brain activation of MCI and Alzheimer’s group, in comparison with healthy aging. Previous studies have proven an activation difference between Alzheimer’s subjects and healthy aging. They showed that resting-state activation network in PCC region of the brain in AD group was smaller than that of Healthy Aging.^15^ Consequently, since activation size and intensity in the presented control group was significantly greater than that of AD group, the same results with the previous studies have reached. Moreover a group of MCI was added, whose results showed that an activation strength has been obtained between that of control and AD groups, in both activation cluster size and intensity.

As mentioned before, MCI is a transition stage between the cognitive impairments of healthy aging subjects and the more serious problems caused by Alzheimer’s disease.^9^ It is believed that MCI is not as severe as Alzheimer’s, and is not as normal as healthy aging either. It can be concluded that in MCI, impairments related to Alzheimer’s has been started to appear, but are not completed yet. It can also be concluded from the present results that PCC region in MCI group shows moderate activation during resting-state, not as powerful as healthy aging, and not as weak as Alzheimer’s, in both cluster size and intensity. Therefore, functional MRI can be used to differentiate MCI, in a transition stage, from AD or healthy aging.

## Conclusions

Although it has already been proved that Alzheimer’s causes some impairments in different brain functions, it is still unclear how these impairments appear. The question here is that whether these mental problems appear suddenly or gradually. To study the disease development, one may investigate MCI, which is the stage between healthy aging and AD. Evaluating activation patterns in healthy aging, MCI and AD groups simultaneously reveals the gradual appearance of brain impairments during the course of the disease development.

Consequently, although it was already proved that fMRI is able to diagnose Alzheimer’s disease, it was showed that fMRI can also be used to predict this disease. This is important due to the fact that most MCI patients are in the pre-stage of AD and at least 20% of them convert to Alzheimer’s each year.^29^
